# Enhanced Skin-Protective Effects of a Novel *Centella asiatica* Variety (BT-Care) Cultivated for 75 Days via Modulation of Antioxidant Defense, Collagen Synthesis, and Skin Barrier Function

**DOI:** 10.4014/jmb.2504.04036

**Published:** 2025-07-14

**Authors:** Kayoung Ko, Ga Yeong Cheon, Yeon Ji Ha, Ye Rin Ko, Jeong Hwan Kim, Daebang Seo, Sook Young Park, Bongkyu Lee, Ki-Bae Hong

**Affiliations:** 1 Department of Food Science and Nutrition, Jeju National University, Jeju 63243, Republic of Korea; 2 ASKBASE BYoungPool R&D Team, Jeju 63309, Republic of Korea; 3 Human Interface Media Center, Jeju National University, Jeju 63243, Republic of Korea; 4 Department of Data Science, Jeju National University, Jeju 63243, Republic of Korea

**Keywords:** *Centella asiatica*, collagen, cultivation, keratinocyte, skin

## Abstract

*Centella asiatica* L. Urban (CA) is traditionally used in skin wound healing, and stable cultivation-related studies are currently being conducted to meet the growing demand in the food, cosmetics, and pharmaceutical industries. This study aimed to analyze the growth rate and target bioactive compound levels of a novel variety, giant CA (BT-care), according to cultivation period, and to compare the effects of extracts harvested at specific cultivation periods on skin health in human keratinocytes with those of general CA. Although yields increased with prolonged BT-care cultivation, the levels of madecassoside, asiaticoside, total polyphenols and radical scavenging activity were significantly higher at 75 days post-transplantation. Compared to general CA, BT-care extracts obtained at day 75 significantly enhanced glutathione peroxidase expression among antioxidant-associated genes in ultraviolet B (UVB)-irradiated HaCaT cells. In addition, BT-care dose-dependently upregulated gene expression involved in collagen synthesis, wrinkle prevention, and skin barrier reinforcement. Furthermore, the high-concentration of BT-care significantly reduced the phosphorylation of c-JUN NH_2_-terminal protein kinase and p38 mitogen-activated protein kinases. After 75 days of cultivation, BT-care significantly modulated protein levels associated with skin homeostasis, recovery, collagen synthesis, and skin barrier-related gene expression, which may be attributed to the elevated levels of active compounds. These findings indicate that BT-care may serve as a promising resource for food, cosmetic, and pharmaceutical applications aimed at promoting skin health.

## Introduction

*Centella asiatica* L. Urban (CA) is a perennial herb of the Apiaceae family that is widely distributed across tropical and subtropical regions worldwide [1]. Traditionally, CA has been used to treat various mild and chronic mental and physical disorders based on empirical knowledge and folk medicine practices [2]. Among the numerous active compounds, the pentacyclic triterpene glycosides (madecassoside and asiaticoside) and their aglycones (madecassic acid and asiatic acid) have been reported to exhibit notable therapeutic properties and pharmacological activities [3]. In addition, CA extracts contain a wide range of bioactive components, including sterols, flavonoids, essential fatty acids, phytosterols, and polyphenols [4].

Scientific evidence supports the traditional use of CA in traditional medicine, highlighting its antioxidant, antinociceptive, and anti-inflammatory properties, as well as its involvement in immune regulation and the management of cardiovascular, neurological, and dermatological disorders [5, 6]. Following oral administration, madecassoside and asiaticoside are converted to their respective aglycones in the intestine and are absorbed into the stomach, liver, kidney, brain, and skin within minutes, where they exert various pharmacological and biological activities [7-9]. In particular, CA extracts and their pentacyclic triterpenes have been reported to be effective against acne, burn, atopic dermatitis, and wounds by modulating the nuclear factor kappa B (NF-κB) and mitogen-activated protein kinase (MAPK) signaling pathways through regulation of inflammatory responses and oxidative stress in both *in vitro* and *in vivo* models [10].

Wrinkle formation and reduced antioxidant activity are significant health indicators associated with aging and skin appearance. Skin aging is classified into intrinsic and extrinsic types based on the underlying factors, with ultraviolet (UV) irradiation-induced oxidative stress contributing to the generation of excessive free radicals. Additionally, elevated activities of matrix metalloproteinase-2 (MMP-2) and hyaluronidase promote the degradation of collagen and hyaluronic acid, respectively, leading to structural damage in both the epidermis and dermis [11]. The barrier function of the skin is maintained by epidermal homeostasis, and the cornified envelope—composed of cross-linked structural proteins such as involucrin (IVL) and filaggrin (FLG)—plays a significant role in protecting the skin from environmental damage and preventing transepidermal water loss within keratinocytes [12, 13]. Furthermore, UV-irradiated skin modulates the production of inflammatory cytokines as part of defense mechanism, thereby influencing overall skin conditions. Numerous natural materials have been utilized in the food and cosmetic industries to protect epidermal and dermal cells from external stimuli, and ongoing research continues to explore bioactive materials through diverse scientific approaches.

CA extracts, which contain various bioactive compounds, have been used to treat wounds, burns, and other minor skin injuries by stimulating collagen production and tissue repair. Currently, CA is applied in the cosmeceutical, pharmaceutical, and food industries in the form of dermal wound care creams, functional foods, and beverages. However, the concentration of active constituents, including triterpenes, in CA varies depending on factors such as geographical origin, genetics, environmental conditions, and cultivation practices [1]. Although CA is traditionally consumed as a medicinal plant across different cultures, it is predominantly harvested from wild populations. Therefore, research is needed to select CA varieties with high productivity and optimal levels of active compounds, as well as to develop improved cultivars. In addition, given CA’s reliance on wild populations and the significant influence of environmental factors on yield and compound content, further studies should focus on identifying appropriate cultivation regions and determining optimal growth periods.

This study aimed to analyze the bioactivity of giant CA (BT-care), a valuable cultivar characterized by larger leaves and stems and higher yield compared to general CA. Although BT-care exhibits greater biomass and elevated levels of key active compounds, including madecassoside and asiaticoside, its effects on skin health parameters such as whitening, wrinkle reduction, and moisturization have not yet been investigated. Therefore, this study compared BT-care harvested at the optimal cultivation period with general CA to assess their regulatory effects on enzymes and signaling pathways involved in skin health using an *in vitro* keratinocyte model, with the goal of confirming the potential applicability of BT-care in skin health-related industries.

## Materials and Methods

### Materials

Dried CA and BT-care leaves used in the analysis were provided by ASKBASE Co. (Republic of Korea). ASKBASE Co., established a cultivation facility equipped with an automatic water level control system for the beds, a sprinkler system, an automated external vinyl opening and closing system responsive to temperature and rainfall, a UV-blocking system, and a heating and cooling system to optimize BT-care growth conditions. For extraction, 750 ml of lava seawater was added to 50 g of the crushed plant material harvested from this cultivation system (w:v = 1:25) and subjected to reflux at 80°C for 24 h. After extraction, the sample was freeze-dried (FDTE-8012, Operon, Republic of Korea) at -80°C for four days and subsequently stored at -20°C until use. Standard madecassoside (≥95.0%) and asiaticoside (≥98.5%) were purchased from Sigma (Sigma Aldrich, USA).

### Triterpene Analysis

Analysis for madecassoside and asiaticoside contents was performed by high performance liquid chromatography (HPLC) according to the manufacturer’s protocol, and all the experiments were triplicated. A 0.1 g sample was dissolved in 5 ml of methanol (Sigma-Aldrich) using an ultrasonic extractor (UCP-20, Jeio Tech., Republic of Korea) for 30 min (Frequency: 50 kHz; temperature: 30°C; power: 500W), followed by purification through a 0.45 μm polytetrafluoroethylene (PTFE) syringe filter (Whatman, UK), and the resulting solution was used for analysis. Sample aliquots of 10 μl were injected into the Nexera lite HPLC system (Shimadzu, Japan), consisting of the Shim-pack GIS C18 column (4.6 mm × 250 mm, Shimadzu), a column heater (30°C), and a photodiode array (PDA) detector (SPD-M40, Shimadzu).

### Assay of Polyphenol, Flavonoid and Radical Scavenging Activity

Total polyphenol content (TPC) was quantified using the Folin-Ciocalteu method, while total flavonoid content (TFC) was determined using the p-dimethylaminocinnamaldehyde (DMACA) method [14, 15]. A standard curve was constructed using gallic acid and catechin to quantify the TPC and TFC of the sample. The radical scavenging activity of the samples was determined using the 2,2'-azino-bis(3-ethylbenzothiazoline-6-sulfonic acid) (ABTS) and 1,1-diphenyl-2-picrylhydrazyl (DPPH) assays according to the method described previously [16, 17]. The percentage of radical scavenging activity was calculated using the following formula: % scavenging activity = (A_control_ - A_sample_)/A_control_ × 100. The absorbance of each analysis was measured using an Infinite 200 PRO microplate reader (Tecan Group Ltd., Switzerland).

### HaCaT Cell Viability

Human-derived keratinocyte HaCaT cells were obtained from Korea Cell Line Bank (Republic of Korea) and maintained in Dulbecco’s Modiﬁed Eagle’s Medium (DMEM; LM001-05, Welgene, Republic of Korea) supplemented with 10% fetal bovine serum (FBS; S101-01, Welgene) and 1% penicillin-streptomycin (LS202-02, Welgene) at 37°C in a humidified atmosphere containing 5% CO_2_. For cell viability assessment, the Quanti-MAX WST-8 cell viability assay kit (Biomax, Republic of Korea) was emplyed. HaCaT cells were seeded in 96-well plates at a density of 1 × 10^5^ cells/ml and incubated for 24 h. The cells were then treated with medium containing CA or BT-care extracts at different concentrations (12.5, 25, 50, 100, 200, and 400 μg/ml) for 24 h. Following treatments, he medium was replaced with DPBS (LB001-02, Welgene), and the cells were exposed to UVB radiation (30 mJ/cm²) using a UV lamp (G15T82, Sankyo Denki, Japan). Post-irradiation, the cells were cultured in extract-containing medium for an additional 24 h. The medium was then removed, cells were washed with DPBS, and 100 μl of WST-8 solution was added to each well. Absorbance was measured at 450 nm using a microplate reader to determine cell viability.

### Quantitative Real-Time Polymerase Chain Reaction (qRT-PCR)

HaCaT cells were seeded at a density of 1 × 10^5^ cells/ml in 6-well plates and treated with medium containing CA or BT-care extracts at different concentrations (25, 50, and 100 μg/ml). The cells were subjected to UVB irradiation (30 mJ/cm²) and subsequently incubated for 24 h in medium containing the same extract concentration. Total RNA was isolated from cells using RNAzol reagent (Invitrogen, USA), and cDNA was synthesized using the SuperScript III Reverse Transcriptase kit (Invitrogen) according to the manufacturer's instructions. Target gene expression was analyzed using TaqMan PCR Master Mix (Applied Biosystems, USA) and StepOne plus Real-Time PCR system (Applied Biosystems). Glyceraldehyde-3-phosphate dehydrogenase (GAPDH) was used as the endogenous genes. The target genes used in the Human model are as follows: superoxide dismutase (SOD 1, NM_000454.4), catalase (CAT, NM_001752.3), glutathione peroxidase (GPX, NM_001329455.2), collagen type 2 alpha 1 (COL2a1, NM_001844.4), matrix metalloproteinases (MMP 2, NM_001127891.2; MMP 3, NM_002422.4; MMP 12, NM_002426.5), tissue inhibitor of metalloproteinases (TIMP 1, NM_003254.2; TIMP 2, NM_003255.4), interlukin-1 alpha (IL-1α, NM_000575.4), IL-6 (NM_000600.4), tumor necrosis factor-alpha (TNF-α, NM_001270949.1), involucrin (IVL, NM_005547.2), filaggrin (FLG, NM_002016.1), and GAPDH, NM_001256799.2.

### Western Blot Analysis

HaCaT cells (5 × 10^5^ cells/ml) were seeded in 6-well plates and treated with extracts at concentrations of 25, 50, and 100 μg/ml for 24 h. Following treatment, cells were irradiated with UVB (30 mJ/cm²) and cultured for an additional 24 h in extract-containing medium. Cellular proteins were extracted using RIPA buffer (Thermo Scientific Fisher, USA) supplemented with phosphatase inhibitor cocktail 2 (Sigma-Aldrich) and protease inhibitor (Sigma-Aldrich). Protein concentrations were quantified using a BCA protein assay kit (Thermo Scientific Fisher). For protein separation, 10 μg of total protein was resolved on a 12% SDS-polyacrylamide gel (SDS-PAGE) at 135 V for 90 min and transferred onto a polyvinylidene fluoride (PVDF) membrane at 100 V for 120 min. The membranes were blocked for 1 h with 5% skim milk or 5% bovine serum albumin (BSA) in TBST (Tris-buffered saline with 0.1% Tween-20) and subsequently washed. Membranes were incubated overnight at 4°C with primary antibodies (GAPDH #5174, extracellular signal-regulated kinase; ERK #9102, pERK #9101, c-JUN NH_2_-terminal protein kinase; JNK #9252, pJNK #9251, p38 MAPK; p38 #9212, and p-p38 #9211; Cell Signaling Technology, USA) and then incubated with the corresponding secondary antibody (anti-rabbit IgG, #7074, Cell Signaling Technology) for 2 h at room temperature. Protein bands were visualized using the ChemiDoc Imaging System (Bio-Rad, USA), and expression levels were normalized to GAPDH as an internal control.

### Statistical Analysis

Data are presented as mean ± standard deviation (SD). Statistical significance was determined using Tukey’s multiple comparison test and Student’s *t*-test, with SPSS software version 24.0 (SPSS Inc., USA). Statistical significance was set at *p* < 0.05.

## Results

### Characterization of BT-Care based on the Harvest Date

**Fig. 1:**
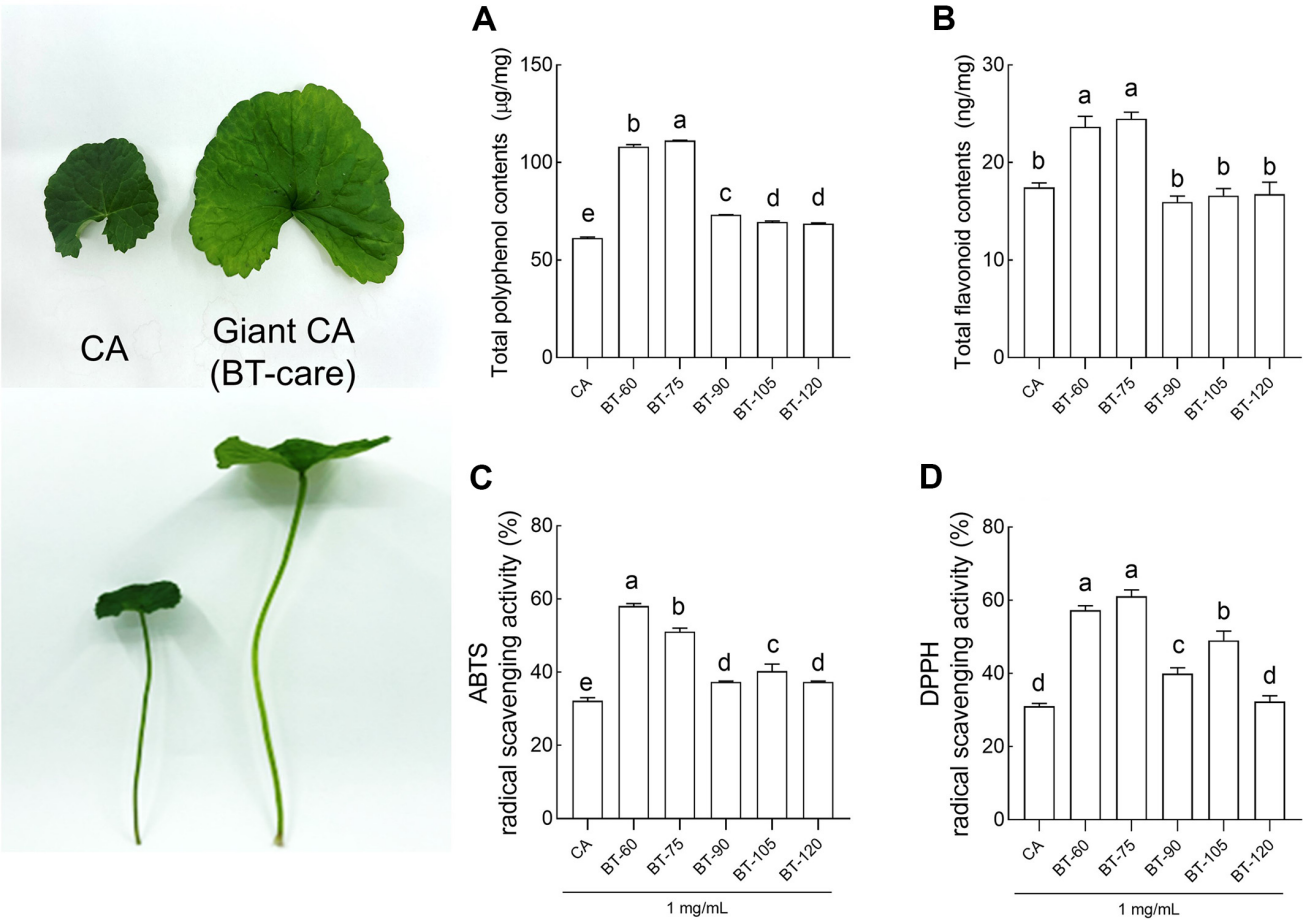
Effects of BT-care by cultivation period on antioxidant activity (A) total polyphenol contents (TPC), (B) total flavonoid contents (TFC) (C) ABTS and (D) DPPH radical scavenging activity. Values are means ± standard deviation (SD); Different letters indicate significant differences between groups at *p* < 0.05 by Tukey's multiple comparison test. CA, *Centella asiatica* extract; BT-care, giant *Centella asiatica*.

When comparing morphological characteristics, BT-care exhibited 2.01-, 1.85-, and 1.53-fold greater leaf length, leaf width, and stem length, respectively, than regular CA (Table S1). Compared to the CA extract, the total polyphenol levels were significantly higher in the BT-care extract on the 75^th^ day of cultivation (Fig. 1A). While morphological alterations in BT-care were observed on days 90, 10, and 120, alterations in levels were reduced during these periods. Regarding total flavonoid levels, BT-care extracts exhibited significantly higher levels at 60 and 75 days compared to CA extracts, with similar levels observed in BT-care extracts harvested after longer cultivation periods (Fig. 1B). The ABTS radical scavenging activity analysis indicated that the BT-care extracts from all cultivation periods were significantly higher than those of the CA extracts, with the highest activity observed at 60 days (Fig. 1C). However, samples harvested after 60 days exhibited a tendency of significant reduction. In the 2,2-diphenyl-1-picrylhydrazyl (DPPH) radical scavenging activity assay, the BT-care extracts at 60 and 75 days of cultivation exhibited a significant increase compared to that of the CA extract, followed by a gradual decline, with no significant difference from CA at 120 days (Fig. 1D). The alterations in madecassoside and asiaticoside levels in BT-care extracts during the cultivation period were analyzed using high-performance liquid chromatography (Table 1 and Fig. S1). High levels of madecassoside and asiaticoside were observed at 60 and 75 days of cultivation. From the 90^th^ to the 120^th^ day, although the size and length of BT-care leaves and stems continued to increase, the levels of madecassoside and asiaticoside declined significantly, showing no positive correlation with vegetative growth.

### Effects of BT-Care on mRNA Expression of Antioxidant Enzymes in UVB-Irradiated HaCaT Cell

**Fig. 2:**
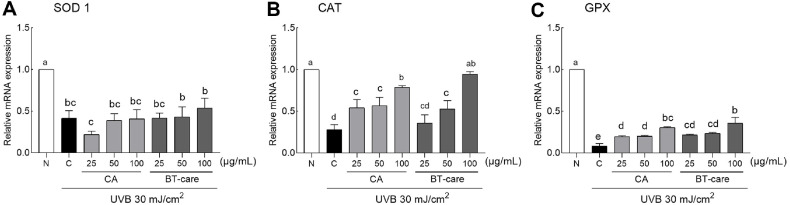
Effects of BT-care extracts on mRNA expression of (A) SOD 1, (B) CAT and (C) GPX in UVBirradiated HaCaT cells. HaCaT cells were treated with 25, 50 and 100 μg/ml concentrations of extracts. Values are the means ± standard deviation (SD) for each group. Different letters indicate significant differences between groups at *p* < 0.05 by Tukey's multiple comparison test. SOD 1, superoxide dismutase 1; CAT, catalase; GPX, glutathione peroxidase; N, normal control group; C, UVB control group; CA, *Centella asiatica* extract; BT-care, giant *Centella asiatica*.

When CA and BT-care extracts were treated with HaCaT cells at concentrations ranging from 12.5–400 μl/ml, cell viability did not reduce to 80 %, even at a high concentration of 400 μl/ml (Fig. S2). Additionally, when CA and BT-care extracts were treated with UVB (30 mJ/cm^2^)-irradiated HaCaT cells, the cell viability significantly increased except for the treatment with 12.5 μl/ml of CA extract (Fig. S3). The concentration of BT-care that significantly increased the viability of UVB-irradiated HaCaT cells was treated at the same levels as CA from 25–100 μl/ml, and the differences in the mRNA expression of antioxidant-related enzymes were analyzed (Fig. 2). When comparing the control group treated only with UVB to the normal group, superoxide dismutase (SOD) expression levels were significantly reduced, with no significant difference from the control group in cells treated with CA and BT-care (Fig. 2A). Although UVB treatment significantly reduced catalase (CAT) mRNA expression levels, these levels were significantly increased across all CA concentrations and middle and high concentrations of BT-care (Fig. 2B). When treated with 100 μl/ml of BT-care, the expression levels were similar to those in the normal group. Additionally, UVB treatment significantly reduced glutathione peroxidase (GPX) mRNA expression levels, whereas CA and BT-care extract treatments significantly increased these levels compared to the control group. Specifically, the high-concentration treatment of CA and BT-care (100 μl/ml) resulted in significantly higher GPX mRNA expression levels (Fig. 2C).

### Effects of BT-Care on mRNA Expression of Extracellular Matrix (ECM)-Related Enzymes and Inflammatory Mediators in UVB-Irradiated HaCaT Cell

**Fig. 3:**
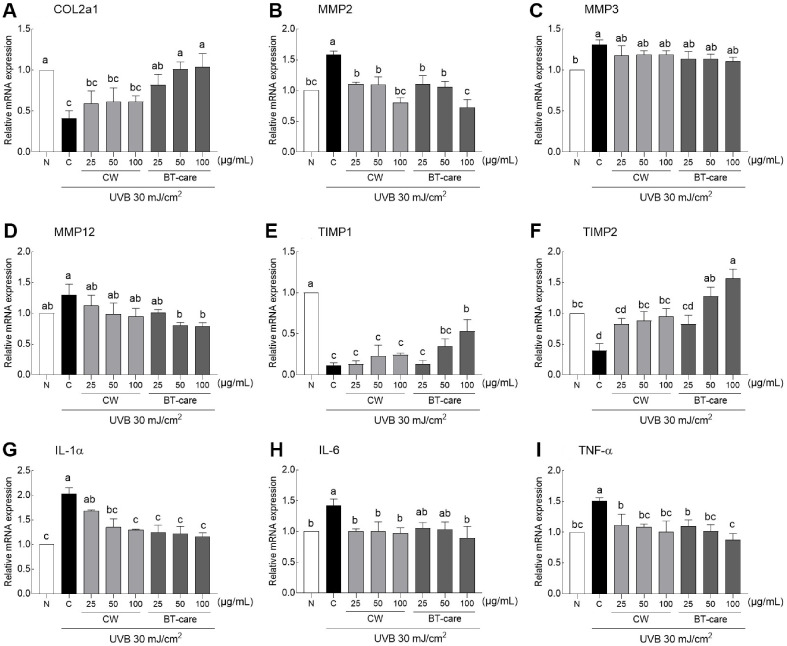
Effects of BT-care extracts on mRNA expression of (A) COL2a1, (B) MMP2, (C) MMP3, (D) MMP12, (E) TIMP1, (F) TIMP2, (G) IL-1α, (H) IL-6 and (I) TNF-α in UVB-irradiated HaCaT cells. HaCaT cells were treated with 25, 50 and 100 μg/ml concentrations of extracts. Values are the means ± standard deviation (SD) for each group. Different letters indicate significant differences between groups at *p* < 0.05 by Tukey's multiple comparison test. COL2a1, collagen type II alpha 1 chain; MMP, Matrix metalloproteinases; TIMP, Tissue Inhibitor of Metallo Proteinases; IL-6 and IL-1α, interleukin-6 and interleukin-1α; N, normal control group; C, UVB control group; CA, *Centella asiatica* extract; BT-care, giant *Centella asiatica*.

UVB irradiation significantly reduced the expression of COL2a1, a collagen synthesis gene in the connective tissue that supports muscles, joints, organs, and skin compared to the expression level in the normal group (Fig. 3A). CA extract treatment did not enhance COL2a1 expression, whereas all concentrations of the BT-care extract significantly increased gene expression. As illustrated in Fig. 3B, MMP2 (gelatinase A) was upregulated by UV irradiation and contributed to ECM protein degradation. Treatment with CA and BT-care extract significantly reduced MMP2 gene expression, with a significant effect observed at high concentrations of BT-care. The expression of MMP3 gene—that degrades collagen, proteoglycans, fibronectin, laminin, and elastin—was increased by UVB irradiation, but did not significantly decrease after CA and BT-care treatment (Fig. 3C). The expression of MMP12 gene that is involved in elastin remodeling, did not significantly differ from that in the normal group after UVB irradiation but was significantly reduced in the 50 and 100 μg/ml BT-care treatments compared to the control group (Fig. 3D). Among the tissue inhibitors of metalloproteinases (TIMPs) that inhibit MMP expression, TIMP1 gene expression that was reduced by UVB irradiation was significantly increased by high-concentration BT-care treatment (Fig. 3E). TIMP2 expression, which was also reduced by UVB irradiation, exhibited significant increases with intermediate and high concentrations of CA and BT-care treatments, and BT-care (100 μg/ml) treatment exhibited a higher expression level than that of the normal group (Fig. 3F). UVB irradiation significantly increased the gene expression of pro-inflammatory cytokines (interleukin-1 alpha [IL-1α], IL-6, and tumor necrosis factor-alpha [TNF-α]) in HaCaT cells, and BT-care treatment more effectively reduced IL-1α expression at all concentrations (Fig. 3G). Compared to the control group, CA extract reduced IL-6 expression to the normal group level more effectively than BT-care, and it was confirmed that BT-care was significantly effective at a high concentration (Fig. 3H). The UVB irradiation-induced increase in TNF-α expression was significantly reduced to the normal level at all concentrations of CA and BT-care treatment (Fig. 3I).

### Effects of BT-Care on mRNA Expression of Barrier-Related Molecules in UVB-Irradiated HaCaT Cell

**Fig. 4:**
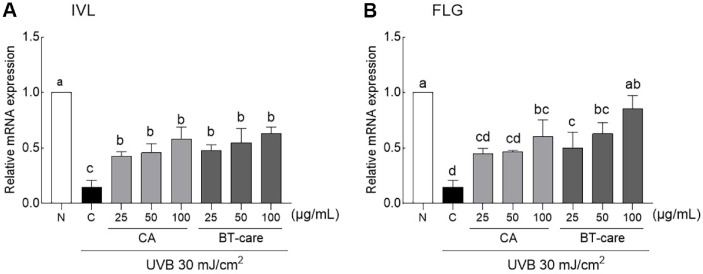
Effects of BT-care extracts on mRNA expression of (A) INV, (B) FLG in UVB-irradiated HaCaT cells. HaCaT cells were treated with 25, 50 and 100 μg/ml concentrations of extracts. Values are the means ± standard deviation (SD) for each group. Different letters indicate significant differences between groups at *p* < 0.05 by Tukey's multiple comparison test. IVL, involucrin; FLG, filaggrin; N, normal control group; C, UVB control group; CA, *Centella asiatica* extract; BT-care, giant *Centella asiatica*.

UVB irradiation significantly reduced the expression of HaCaT intracellular IVL gene that are involved in maintaining the structural stability beneath the cell membrane (Fig. 4A). Treatment with CA and BT-care extract, regardless of concentration, significantly enhanced IVL gene expression, reversing the UVB irradiation-induced reduction. UVB irradiation significantly reduced the expression of FLG, a filament-related gene that binds to keratin fibers of epithelial cells. Treatment with high concentrations of CA significantly increased FLG gene expression, whereas BT-care treatment demonstrated a concentration-dependent increase (Fig. 4B).

### Effects of BT-Care on MAPK Signaling Pathway in UVB-Irradiated HaCaT Cell

**Fig. 5:**
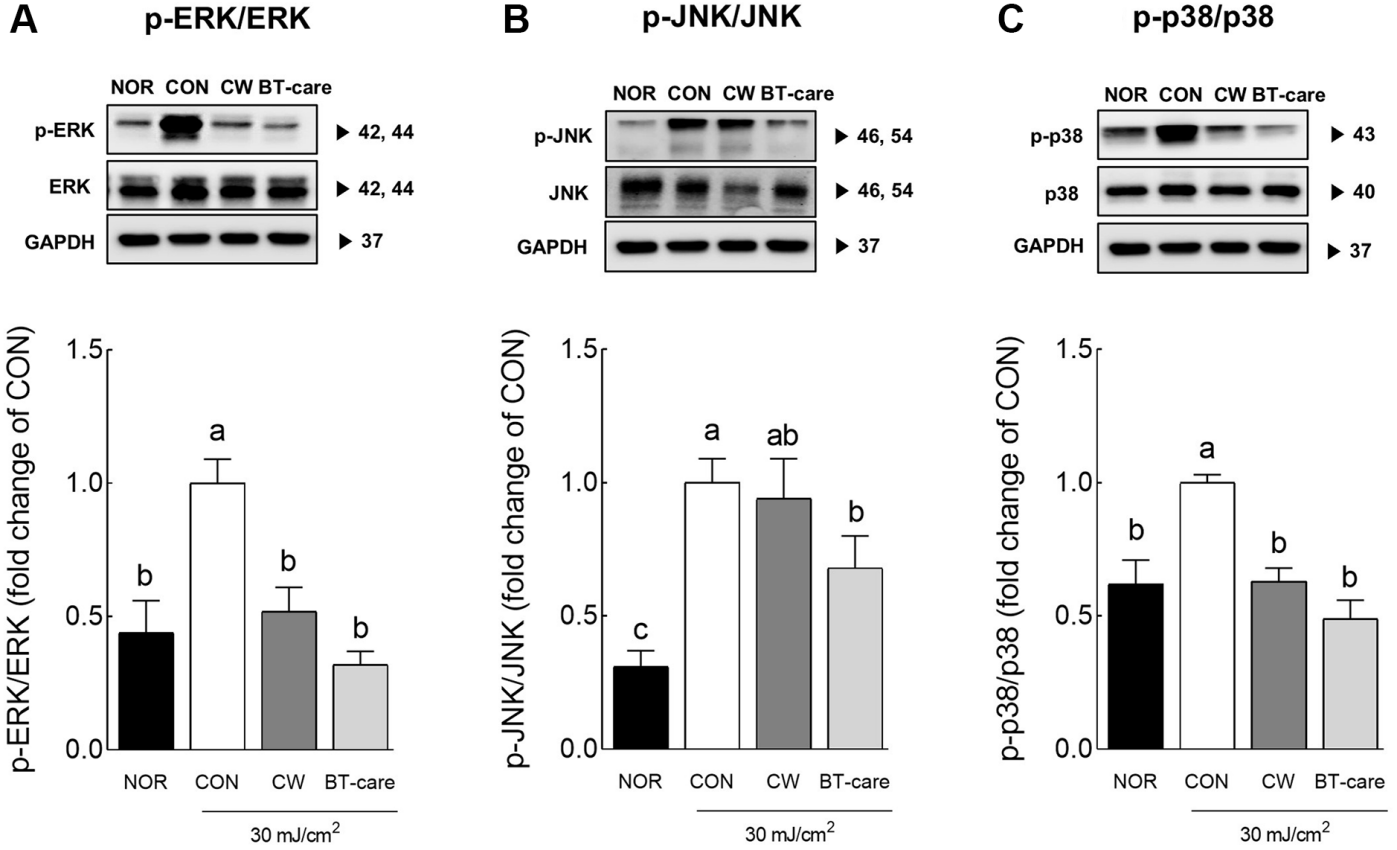
Effects of BT-care extracts on protein expressions of (A) p-ERK/ERK, (B) pJNK/JNK and (C) p-p38/ p38 in UVB-irradiated HaCaT cells. HaCaT cells were treated with 100 μg/ml concentrations of extracts. Values are the means ± standard deviation (SD) for each group. Different letters indicate significant differences between groups at *p* < 0.05 by Tukey's multiple comparison test. ERK, extracellular signal-regulated kinase; JNK, c-JUN NH_2_-terminal protein kinase; p38, p38 mitogen-activated protein kinases. NOR, normal control group; CON, UVB-control group; CA, *Centella asiatica* extract; BT-care, giant *Centella asiatica*.

In a previous experiment, treatment with 100 μl/ml of CA extract effectively reduced phosphorylation of ERK and p-38 to levels similar to those in the normal group (Fig. 5A and 5C). Treatment with BT-care extract at concentrations similar to CA extract phosphorylated JNK, p38, and ERK, significantly reducing the UVB irradiation-induced phosphorylation in the MAPK signaling pathway (Fig. 5A, 5B and 5C).

## Discussion

In this study, the biological activities of a novel CA variety, BT-care, were evaluated with respect to its cultivation period and compared with those of general CA, focusing on skin health-related functions. Our results indicate that the components and antioxidant activity of BT-care vary depending on the harvest date, as shown in Fig. 1 and Table 1. These findings suggest that morphological traits alone, such as increased leaf size during certain cultivation periods, may not fully explain the variation in functional compound levels. As reported in previous studies, an increase in leaf size does not necessarily result in greater accumulation of bioactive compounds. Such divergence between growth and phytochemical accumulation has been demonstrated in *Hypericum perforatum*, where secondary metabolites such as hypericin and catechin showed no positive correlation with biomass under different environmental and cultivation conditions, and in *Moringa oleifera*, where enhanced leaf growth in hydroponic conditions did not always correspond to increased metabolite levels [18, 19]. CA, a high-value medicinal plant used to maintain and facilitate physical and mental health, has been overharvested owing to increasing demand, making commercial cultivation essential for the rapid production of CA elites with optimal concentrations of functional components. Stable CA production considers factors, such as soil type, organic cultivation, and seasonal alterations, with studies reporting the effects of genotype-environment interactions [20, 21]. Ecological niche modeling enables the identification of triterpene level-based chemotypes, mapping potential cultivation areas at large spatial scales, and exploring the effect of climate change on CA production [20, 22]. Among the CA plant parts, the leaves contain the largest amount of pentacyclic triterpenes that varies based on the place of cultivation, harvesting period, and extraction method, such as an ultrasonic-assisted extraction [23, 24]. Jeju Island in the Republic of Korea provides ideal geographic conditions for stable BT-care production (rich in active ingredients) owing to its mild subtropical weather and climate characteristics with high annual average humidity.

As illustrated in Fig. 2, we assessed how BT-care regulates antioxidant-related gene expression in skin cells compared to the general CA. Callus culture of CA in MS medium supplemented with 1-naphthaleneacetic acid, 6-benzylaminopurine, Agargellan, and sucrose exhibited antioxidant activity in the DPPH assay and significantly enhanced the gene expression of antioxidant-related enzymes, such as SOD, CAT, and GPx1 in H_2_O_2_-treated human foreskin fibroblasts [25]. In animal skin flap models, Feng *et al*. [26] verified that the oral administration of asiaticoside (40 mg/kg) regulated the expression of SOD, vascular endothelial growth factor, malondialdehyde, and inflammatory responses, thereby enhancing skin flap viability and function. Additionally, topical application of a gel containing CA transfersomes and bergamot essential oil nanoemulsions to BALB/c mice prevented UB irradiation-induced wrinkle formation and skin erythema and suppressed histological damage by enhancing SOD expression [27]. Triterpenoids in CA enhance the antioxidant defense system, thereby alleviating structural and functional skin alterations resulting from reactive oxygen species (ROS) accumulation-induced mitochondrial damage.

We assessed the effect of BT-care on the levels of ECM-related enzymes and inflammatory mediators in UVB-irradiated HaCaT cells (Fig. 3). Polyphenols, such as flavonoids present in various plant extracts, react with ROS to neutralize free radicals, thereby facilitating skin wound healing and serving as anti-aging agents. In addition to antioxidant-rich triterpene glycosides and phenolics, theoretical analysis indicated that among the flavonoids in CA, castilliferol has higher antioxidant activity than castillicetin [28]. Among the 12 undescribed triterpenoid pentacyclic glycosides in *Herniaria glabra* L., 29-hydroxy-medicagenic acid and herniarinic acid exhibit anti-wrinkle and anti-pigmentation activities by inhibiting collagenase, elastase, and tyrosinase enzymes [29]. Additionally, aloesin and aloin A and B that are contained in *Aloe vera* L., are used for moisturizing, regenerating, and skin erythema, inhibiting melanogenesis and collagen fragmentation by regulating tyrosinase and collagenase activities [30, 31]. Numerous botanical antioxidants, including carotenoids, flavonoids, and polyphenols, exhibit antioxidant effects, UV protection, metal chelation, and enzyme regulation associated with skin conditions. UV irradiation causes ROS accumulation and activates inflammatory responses in the dermal layer, thereby disrupting skin homeostasis and altering ECM proteins, such as collagen, fibronectin, elastin, and proteoglycans. Genome-wide transcriptional profiling revealed that the expression levels of cytokines and chemokines were significantly increased in the human skin tissue after UV irradiation [32]. Additionally, UV irradiation activates inflammasomes, such as the NLR family pyrin domain-containing protein 3, thereby facilitating the secretion of inflammatory factors in human keratinocytes [33]. Inflammatory cytokines (ILs and TNF-α) and ROS enhance the transcription of MMPs through the subsequent activation of cellular and molecular factors and reduce TIMP levels and collagen synthesis in human dermal fibroblasts [34].

In HaCaT keratinocytes, UV irradiation induces stress response regulation, with a reduction in IVL expression, an increase in MMP-1 expression, and the production of cytokines, such as IL-6 and IL-8 [35]. Proteins, such as IVL, FLG, and loricrin (LOR) form the stratum corneum in response to UV irradiation and are involved in skin barrier function and skin moisturizing. Accumulated UV irradiation and ROS reduce the expression of barrier-related molecules and increase the epidermal water loss [36, 37]. Collagen degradation is facilitated by MMPs induced by UV irradiation and ROS accumulation, which forms wrinkles, prevents skin moisture loss, and deteriorates barrier function by reducing stratum corneum proteins. Madecassoside, a major component of CA, enhances skin protection by upregulating the protein levels of LOR, aquaporin-3, IVL, and FLG in HaCaT cells treated with 100 μM madecassoside [38]. An *in vivo* study using SKH-1 hairless mice reported that oral administration of collagen peptides can reduce UV irradiation-induced photoaging by upregulating skin hydration factors (IVL and FLG) and hyaluronic acid synthases (HAS-1 and -2) [39]. Additionally, the regulation of protein expression levels of IVL, FLG, and LOR—indicators of keratinocyte differentiation—was analyzed by treating HaCaT cells with daphnin, daphnetin-8-O-glucoside, daphnetin, rutarensin, isoquercitrin, 7-hydroxy-6-methoxychromone, and daphnoretin, which are the components of *Stella chamaejasme* L. The results indicated that these plant-derived compounds may facilitate skin wound healing and anti-inflammatory activity [40].

UVB irradiation of epidermal keratinocytes facilitates the expression of activator protein-1 (AP-1) and cyclooxygenase-2 (COX-2) while activating the MAPK signaling pathway, thereby mediating various skin diseases. MAPK-activated AP-1 translocates from the cytoplasm to the nucleus, facilitates MMP expression, and inhibits procollagen I expression, which forms type I collagen, thereby inducing the photoaging of the dermal layer [41]. In transgenic mice, p38 inhibition was reported to correlate with COX-2 expression and inhibit UVB irradiation-induced skin cancer proliferation and apoptosis, indicating that p38 can be a major target for pharmacological or non-pharmacological interventions [42]. In HaCaT cells treated with lipopolysaccharide to induce inflammation, CA ethyl acetate extract treatment exhibited anti-inflammatory effects and alleviated psoriasis by inhibiting the activation of the NF-κB and Janus kinase/signal transducer signaling pathways, which regulate cell proliferation, differentiation, apoptosis, and immune responses [43]. Transforming growth factor-β (TGF-β) is involved in fibrotic skin diseases by inducing decapentaplegic (Smad) signaling. Among the triterpenoid compounds of CA, glycosides (madecassoside and asiaticoside) were reported to modulate the TGF-β/Smad signaling pathway and activate skin fibroblasts to enhance collagen synthesis and wound healing compared to metabolites in an ICR mouse experiment [44]. A gel containing asiaticoside, a major component of CA, was assessed in an animal model to facilitate the healing of diabetic skin ulcers by regulating the Wnt/β-catenin signaling pathway that is involved in cell proliferation, apoptosis, and differentiation [45]. In summary, the CA extract and glycosides (madecassoside and asiaticoside) were involved in the regulation of various signaling pathways associated with cell function and exhibited high activity in skin regeneration.

## Conclusion

In conclusion, the novel BT-care variety cultivated for 75 days exhibited significantly enhanced levels of bioactive compounds, including madecassoside, asiaticoside, and polyphenols, and increased radical scavenging activity compared to the levels observed in prolonged cultivation periods. These enhancements corresponded to the enhanced modulation of protein and gene expressions associated with skin homeostasis, collagen synthesis, and skin barrier function in UVB-irradiated HaCaT cells. Under UVB irradiation, BT-care exhibited superior effects over general CA in upregulating antioxidant-related gene expression and reducing protein phosphorylation in the MAPK signaling pathway, specifically JNK and p38. These findings indicate that BT-care, which can be grown at sustainable intervals, has significant potential as a valuable raw material for the food, cosmetic, and pharmaceutical industries, targeting skin health and recovery.

## Supplemental Materials

Supplementary data for this paper are available on-line only at http://jmb.or.kr.



## Figures and Tables

**Table 1:** Madecassoside and asiaticoside contents of giant *Centella asiatica* (BT-care) by cultivation period.

Cultivation period (days)	60	75	90	105	120
Madecassoside (g/kg)	24.26 ± 1.04^a^	25.86 ± 0.94^a^	8.81 ± 0.04^b^	8.92 ± 0.05^b^	7.52 ± 0.04^b^
Asiaticoside (g/kg)	10.28 ± 0.26^b^	12.74 ± 0.21^a^	4.33 ± 0.07^c^	4.01 ± 0.07^cd^	3.88 ± 0.05^d^

Values are the mean ± standard deviation (SD) for each group. Different letters indicate significant differences between groups at *p* < 0.05 by Tukey's multiple comparison test.
