# Supplementary material for: Enhanced Skin-Protective Effects of a Novel Centella asiatica Variety (BT-Care) Cultivated for 75 Days via Modulation of Antioxidant Defense, Collagen Synthesis, and Skin Barrier Function
Source: J Microbiol Biotechnol. 2025 Jul 14;35:e2504036. doi: 10.4014/jmb.2504.04036 (PMC12283260; doi:10.4014/jmb.2504.04036)
Supplement: Supplementary file 1 [file jmb-35-e2504036-supple.pdf]

J. Microbiol. Biotechnol. 2025. 35: e2504036

https://doi.org/10.4014/jmb.2504.04036

Table S1. Morphological characteristics observed in BT-care.

	Leaf length (cm)	Leaf width (cm)	Stem length (cm)
General CA	4.47 ± 0.38	2.33 ± 0.19	17.77 ± 2.13
BT-care	8.99 ± 0.59***	4.29 ± 0.23***	27.00 ± 0.50**

Values are the mean ± standard deviation (SD) for each group. CA, *Centella asiatica* extract; BT-care, giant *Centella asiatica*.

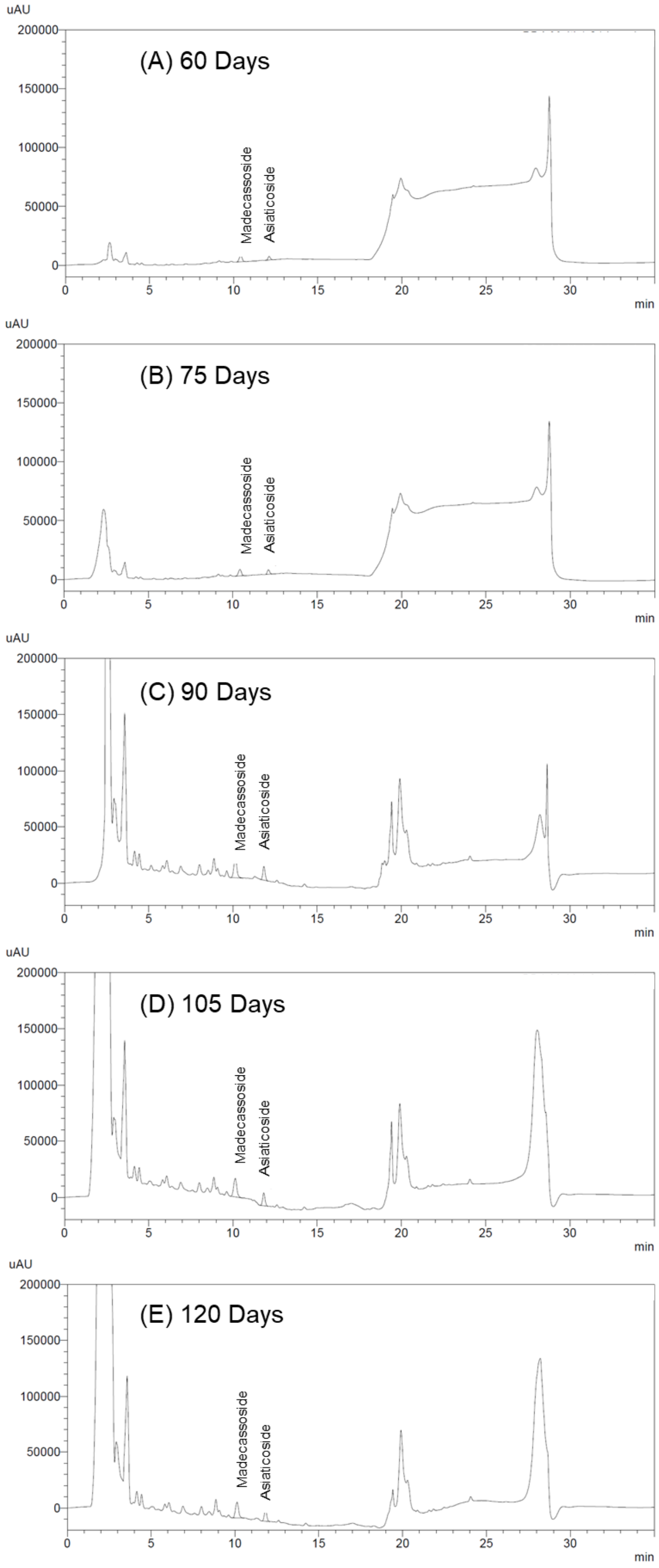

Fig. S1. High-performance liquid chromatography chromatogram of madecassoside and asiaticoside levels in BT-care extracts during the cultivation period.

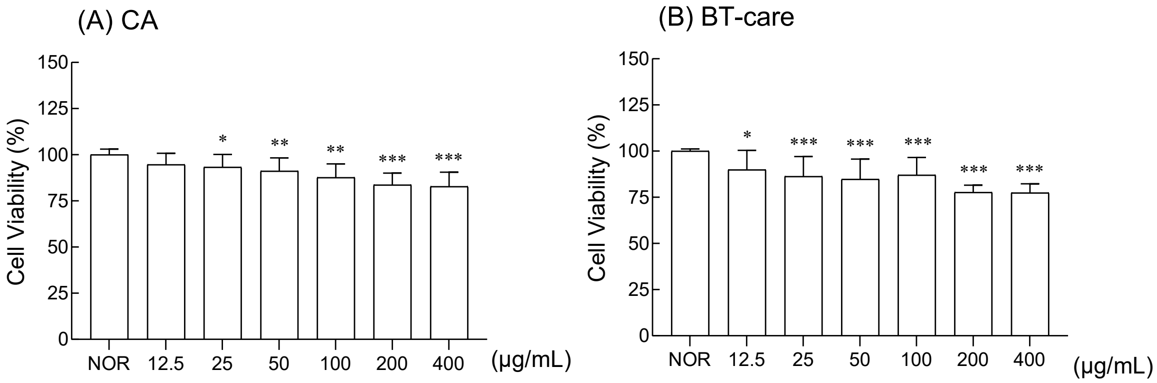

Fig. S2. Effects of BT-care extracts on cell viability in HaCaT cells. HaCaT cells were treated with 12.5, 25, 50, 100, 200 and 400 μg/mL concentrations of extracts. Values are the means ± standard deviation (SD) for each group. Symbols indicate significant differences at $*p<0.05$, $**p<0.01$ and $***p<0.001$ vs. NOR groups using Student's *t*-test. CA, *Centella asiatica* extract; BT-care, giant *Centella asiatica*.

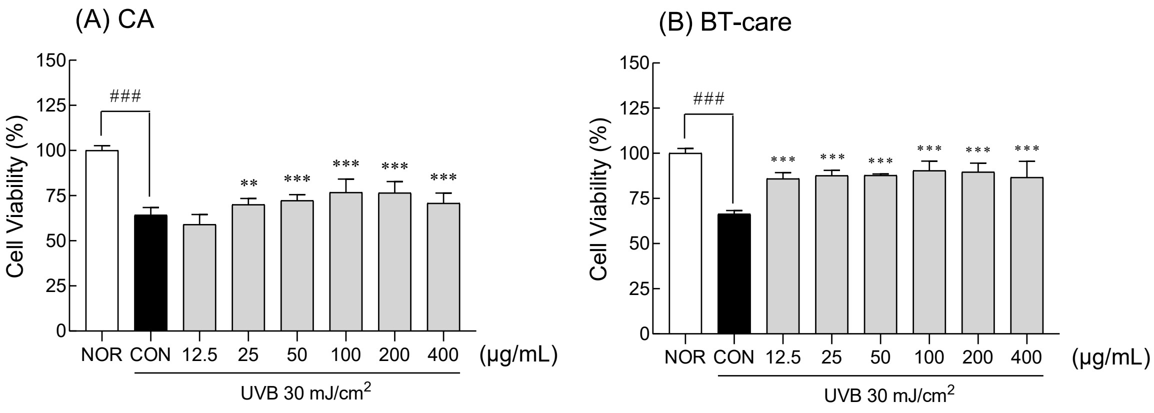

Fig. S3. Effects of BT-care extracts on cell viability in UVB-irradiated HaCaT cells. HaCaT cells were treated with 12.5, 25, 50, 100, 200 and 400 μg/mL concentrations of extracts. Values are the means ± standard deviation (SD) for each group. Symbols indicate significant differences at *p<0.05, **p<0.01 and ***p<0.001 vs. NOR groups using Student's t-test. CA, *Centella asiatica* extract; BT-care, giant *Centella asiatica*.
